# Curcumin Pretreatment Prevents Potassium Dichromate-Induced Hepatotoxicity, Oxidative Stress, Decreased Respiratory Complex I Activity, and Membrane Permeability Transition Pore Opening

**DOI:** 10.1155/2013/424692

**Published:** 2013-07-17

**Authors:** Wylly Ramsés García-Niño, Edilia Tapia, Cecilia Zazueta, Zyanya Lucía Zatarain-Barrón, Rogelio Hernández-Pando, Claudia Cecilia Vega-García, José Pedraza-Chaverrí

**Affiliations:** ^1^Department of Biology, Faculty of Chemistry, National Autonomous University of Mexico (UNAM), University City, 04510 Mexico City, DF, Mexico; ^2^Renal Pathophysiology Laboratory, Department of Nephrology, National Institute of Cardiology “Ignacio Chávez”, 14080 Mexico City, DF, Mexico; ^3^Department of Cardiovascular Biomedicine, National Institute of Cardiology “Ignacio Chávez”, 14080 Mexico City, DF, Mexico; ^4^Experimental Pathology Section, National Institute of Medical Sciences and Nutrition “Salvador Zubirán”, 14000 Mexico City, DF, Mexico; ^5^Department of Preclinical Toxicology, National Polytechnic Institute, 14740 Mexico City, DF, Mexico

## Abstract

Curcumin is a polyphenol derived from turmeric with recognized antioxidant properties. Hexavalent chromium is an environmental toxic and carcinogen compound that induces oxidative stress. The objective of this study was to evaluate the potential protective effect of curcumin on the hepatic damage generated by potassium dichromate (K_2_Cr_2_O_7_) in rats. Animals were pretreated daily by 9-10 days with curcumin (400 mg/kg b.w.) before the injection of a single intraperitoneal of K_2_Cr_2_O_7_ (15 mg/kg b.w.). Groups of animals were sacrificed 24 and 48 h later. K_2_Cr_2_O_7_-induced damage to the liver was evident by histological alterations and increase in the liver weight and in the activity of alanine aminotransferase, aspartate aminotransferase, lactate dehydrogenase, and alkaline phosphatase in plasma. In addition, K_2_Cr_2_O_7_ induced oxidative damage in liver and isolated mitochondria, which was evident by the increase in the content of malondialdehyde and protein carbonyl and decrease in the glutathione content and in the activity of several antioxidant enzymes. Moreover, K_2_Cr_2_O_7_ induced decrease in mitochondrial oxygen consumption, in the activity of respiratory complex I, and permeability transition pore opening. All the above-mentioned alterations were prevented by curcumin pretreatment. The beneficial effects of curcumin against K_2_Cr_2_O_7_-induced liver oxidative damage were associated with prevention of mitochondrial dysfunction.

## 1. Introduction

Curcumin or diferuloylmethane (1,7-bis[4-hydroxy-3-methoxyphenyl]-1,6-heptadiene-3,5-dione) is a hydrophobic polyphenol derived from turmeric: the rhizome of the herb *Curcuma longa* [1]. Traditionally, turmeric has been used in therapeutic preparations for various ailments in different parts of the world [2]. At present, turmeric is used as a dietary spice, by the food industry as additive, flavouring, preservative and as colouring agent in foods and textiles [3, 4]. Curcumin is a major component of turmeric and it has been shown to exhibit antioxidant [5], antimicrobial [6], anti-inflammatory [7], and anticarcinogenic [8] activities. 

The antihepatotoxic effects of curcumin against chemically induced hepatic damage are well documented, and they have been attributed to its intrinsic antioxidant properties [9]. Thus, curcumin has shown to protect liver against hepatic injury and fibrogenesis by suppressing hepatic inflammation [10], attenuating hepatic oxidative stress [11, 12], increasing expression of the xenobiotic detoxifying enzymes such as superoxide dismutase (SOD), catalase (CAT), glutathione peroxidase (GPx), glutathione-*S*-transferase (GST), glutathione reductase (GR), and NAD(P)H:quinone oxidoreductase [5, 13, 14], inhibiting hepatic stellate cells activation [15–17], and supporting the mitochondrial function [18].

On the other hand, chromium exists in several oxidation states, being hexavalent chromium [Cr(VI)] and trivalent chromium [Cr(III)] the most stable forms. Cr(III) is predominantly present in the environment and in salts used as micronutrients and dietary supplements [19]. Cr(VI) salts such as potassium dichromate (K_2_Cr_2_O_7_) or chromic acid are widely used in leather, chrome-plating, and dye-producing industries [20, 21]. Occupational and environmental exposure to Cr(VI)-containing compounds is known to be toxic, mutagenic, and carcinogenic to human beings and diverse animals [22–24], leading to serious damage to the kidneys [25, 26], liver [27, 28], lungs [29, 30], skin [31], and other vital organs [32–34].

Cr(VI) is generally considered to be the toxic form, which can efficiently penetrate anionic channels in cellular membranes [35]. Inside cells, Cr(VI) is reduced through reactive intermediates Cr(V), Cr(IV), and to the more stable Cr(III) by cellular reductants such as glutathione, cysteine, ascorbic acid, and riboflavin and NADPH-dependent flavoenzymes [36]. In fact, the redox couples Cr(VI)/(V), Cr(V)/(IV), and Cr(III)/(II) have been shown to serve as cyclical electron donors in a Fenton-like reaction, which generates reactive oxygen species (ROS) leading to genomic DNA damage and oxidative deterioration of lipids and proteins [37]. 

Liver is an organ capable of being injured by Cr(VI), and it has been demonstrated that the exposition to K_2_Cr_2_O_7_ induces hepatotoxicity associated to increased ROS levels [38], lipid peroxidation [39, 40], inhibition of antioxidant enzymes [41, 42], structural tissue injury [43, 44], and mitochondrial damage [45] including impaired mitochondrial bioenergetics [46, 47]. Natural and synthetic antioxidants have been reported to ameliorate or prevent K_2_Cr_2_O_7_-induced hepatotoxicity [42, 48, 49]. In this context, Molina-Jijón et al. [50] have recently shown that curcumin pretreatment has a protective role in K_2_Cr_2_O_7_-induced nephrotoxicity, and Chandra et al. [51] demonstrated protective effects of curcumin against K_2_Cr_2_O_7_ in male reproductive system. However, to our knowledge, the potential antihepatotoxic protective effect of curcumin on K_2_Cr_2_O_7_-induced hepatotoxicity has not been explored. The purpose of this study was to explore the potential protective effect of curcumin pretreatment against the K_2_Cr_2_O_7_-induced hepatotoxicity, oxidative stress, and mitochondrial dysfunction.

## 2. Materials and Methods

### 2.1. Reagents and Antibodies

Curcumin, K_2_Cr_2_O_7_, bovine serum albumin, butylated hydroxytoluene (BHT), 1-methyl-2-phenylindole, tetramethoxypropane, streptomycin sulfate, guanidine hydrochloride, 2,4-dinitrophenylhydrazine (DNPH), xanthine, xanthine oxidase, nitroblue tetrazolium (NBT), glutathione reduced form (GSH), glutathione oxidized form (GSSG), GR, GST, 1-chloro-2,4-dinitrobenzene (CDNB), dimethyl sulfoxide (DMSO), nicotinamide adenine dinucleotide phosphate reduced form (NADPH), N-(2-hydroxyethyl) piperazine-N′-(2-ethanesulfonic acid) (HEPES), adenosine diphosphate (ADP), potassium succinate, rotenone, sodium glutamate, sodium malate, carbonyl cyanide m-chlorophenylhydrazone (CCCP), decylubiquinone, nicotinamide adenine dinucleotide reduced form (NADH), ethylene glycol tetraacetic acid (EGTA), 3-(N-morpholino) propanesulfonic acid (MOPS), potassium cyanide (KCN), antimycin A, safranin O, sucrose, and paraformaldehyde were purchased from Sigma-Aldrich (St. Louis, MO, USA). Commercial kits to measure alanine aminotransferase (ALT), aspartate aminotransferase (AST), lactate dehydrogenase (LDH), and alkaline phosphatase (ALP) were from ELITechGroup (Sées, France). Monochlorobimane was purchased from Fluka (Schnelldorf, Germany). Potassium phosphate monobasic (KH_2_PO_4_), sodium phosphate dibasic (Na_2_HPO_4_), trichloroacetic acid (TCA), hydrogen peroxide (H_2_O_2_), methanol, high-performance liquid chromatography- (HPLC-) grade acetonitrile, and ethyl acetate were acquired from J. T. Baker (Xalostoc, Edo. Mex, México). Tris, acrylamide and bis N,N′ methylene bis acrylamide were purchased from Bio-Rad Laboratories (Hercules, CA, USA). Lauryl sulfate sodium salt (SDS) and calcium chloride were acquired from Research Organics, Inc. (Cleveland, OH, USA). Arsenazo III was purchased from ICN Biomedicals Inc. (Aurora, OH, USA). Aminoacetic acid was obtained from Química Meyer (Mexico, DF, Mexico). Cyclosporine A (CsA) was purchased from Enzo Life Sciences (Farmingdale, NY, USA). Anti-cytochrome c [7H8.2C12] (ab13575) antibody was acquired from Abcam (Cambridge, MA, USA), anti-adenine nucleotide translocator (ANT) 1/2 (N-19) (sc-9299) and rabbit anti-mouse IgG-horseradish peroxidase (HRP, sc-358914) antibodies were purchased from Santa Cruz Biotechnology (Santa Cruz, CA, USA). All other reagents and chemicals used were of the highest grade of purity commercially available. 

### 2.2. Experimental Design

Male Wistar rats weighing 150–200 g were used along the study. Curcumin was suspended in 0.5% carboxymethylcellulose and was given by oral gavage a dose of 400 mg/kg [50], and K_2_Cr_2_O_7_ was dissolved in saline solution and given via intraperitoneal (i.p.) at a dose of 15 mg/kg [52]. Six groups of rats were studied (*n* = 8/group). (1) Control, injected via i.p. with isotonic saline solution. (2) Curcumin was given daily for 10 days. (3) K_2_Cr_2_O_7_ (24 h), rats received a single injection of K_2_Cr_2_O_7_ on day 10 and they were sacrificed 24 h later. (4) CUR-K_2_Cr_2_O_7_ (24 h), curcumin was given daily for 10 days and K_2_Cr_2_O_7_ was injected on day 10; rats were sacrificed 24 h later. (5) K_2_Cr_2_O_7_ (48 h), rats were injected with a single injection of K_2_Cr_2_O_7_ on day 9 and they were sacrificed 48 h later. (6) CUR-K_2_Cr_2_O_7_ (48 h), curcumin was administered for 10 days and K_2_Cr_2_O_7_ was injected on day 9; rats were sacrificed 48 h later. Animals were anesthetized and blood was obtained via abdominal aorta at room temperature on day 11. Blood plasma was separated and stored at 4°C until the activity of ALT, AST, LDH, and ALP was measured using commercial kits. Livers were dissected out, cleaned, and weighted, obtaining samples for histological analyses and for measurement of oxidative stress markers and activity of the antioxidant enzymes SOD, CAT, GPx, GR, and GST. Liver samples were removed to isolate mitochondria in order to determine oxidative stress markers, activity of antioxidant enzymes, oxygen consumption and the activity of NADH:ubiquinone oxidoreductase (respiratory complex I), mitochondrial permeability transition, and cytochrome (cyt c) release. All procedures were made to minimize animal suffering and were approved by the Local Ethical Committee (FQ/CICUAL/036/12). Experimental protocols followed the guidelines of Norma Oficial Mexicana for the use and care of laboratory animals (NOM-062-ZOO-1999) and for disposal of biological residues (NOM-087-SEMARNAT-SSA1-2002).

### 2.3. Histological Studies

Liver slices of 0.5 cm width were fixed by immersion in 4% paraformaldehyde, dehydrated, and embedded in paraffin. Thin sections of 3 *μ*m were stained with hematoxylin and eosin and were examined under light microscope Leica (Cambridge, UK) [53]. The histological profiles of seven fields 100X randomly selected per rat (3-4 rats per group) were recorded, the number of necrotic hepatocytes (shrinking cells with condensed acidophilic cytoplasm and pyknotic or fragmented nucleus), was counted (*n* = 500–800 hepatocytes), and the percentage of damaged cells was obtained.

### 2.4. Markers of Oxidative Damage and Activity of Antioxidant Enzymes in Liver Homogenates

Liver was homogenized in a Brinkmann Polytron Model PT 2000 (Westbury, NY, USA) in cold potassium phosphate buffer. The homogenates were centrifuged and the supernatant was separated to measure oxidative stress markers and the activity of antioxidant enzymes. Protein concentration was measured according to the method described by Lowry et al. [54]. The content of malondialdehyde (MDA), an important toxic byproduct of lipid peroxidation, was measured by the reaction with 1-methyl-2-phenylindole, according to Chirino et al. [55]. Protein carbonyl content, a relatively stable marker of protein oxidation by ROS, was measured by their reactivity with DNPH to form protein hydrazones, as previously described by Maldonado et al. [56]. GSH content was evaluated by following the formation of a fluorescent adduct between GSH and monochlorobimane in a reaction catalyzed by GST [57]. CAT activity was assayed by a method based on the decomposition of H_2_O_2_ by CAT contained in the samples [58]. SOD activity was assayed by measuring the inhibition of NBT reduction to formazan at 560 nm [59]. GPx activity was assayed spectrophotometrically at 340 nm in a mixture assay containing GSH, H_2_O_2_, GR, and NADPH [60]. GR activity was assayed by using GSSG as substrate and measuring the disappearance of NADPH at 340 nm [61]. GST activity was assayed in a mixture containing GSH and CDNB [62]. 

### 2.5. Studies in Isolated Mitochondria

Liver was removed from rats, washed, and minced in isolation buffer before being homogenized. Mitochondria were obtained by differential centrifugation, and the protein content was measured [63]. Markers of oxidative damage and activity of antioxidant enzymes were measured as previously described in Section 2.4. Oxygen consumption was measured using a Clark type oxygen electrode (Yellow Springs Instruments, Yellow Springs, OH, USA). State 4 respiration was evaluated in the presence of succinate plus rotenone or with sodium glutamate and sodium malate. State 3 respiration was stimulated by the addition of ADP. Respiratory control index (RCI) was calculated as the ratio state 3/state 4. Uncoupled respiration was measured by adding CCCP; phosphorylation efficiency (ADP/O ratio) was calculated from the added amount of ADP and total amount of oxygen consumed during state 3 [64]. The activity of the respiratory complex I was measured by following the decrease in absorbance due to oxidation of NADH to NAD^+^ at 340 nm in an assay mixture containing decylubiquinone antimycin A, KCN, and of mitochondrial protein [65]. Permeability transition pore (PTP) opening was evaluated by measuring swelling which was assessed by changes in absorbance of the suspension at 540 nm, after the addition of 50 *μ*M Ca^2+^ [66]. Membrane potential dissipation was evaluated by safranin O absorbance changes at 525–575 nm; the reaction was initiated by adding 50 *μ*M Ca^2+^ [67]. Ca^2+^ retention was determined by the arsenazo III absorbance changes at 675–685 nm, after the addition of 50 *μ*M Ca^2+^ [68]. These assays were effectuated in the presence or absence of CsA. To assess cytochrome c (cyt c) release, mitochondria were incubated with 50 *μ*M Ca^2+^ with or without CsA for 10 min and pelleted by centrifugation. Released cyt c in the supernatant fractions and retained cyt c in pellets were analyzed by immunoblotting with anti-cyt c (1 : 2,500) as described by Zazueta et al. [69]. Adenine nucleotide translocator (ANT, 1 : 1,000) content was determined as the loading marker. Cyt c and ANT levels were determined by densitometric analysis using the Image Lite Version 3.1.4 software from LI-COR Biosciences (Lincoln, NE, USA).

### 2.6. Statistical Analysis

Results were expressed as mean ± standard error of the mean (SEM). Data were analyzed by one-way ANOVA followed by Bonferroni's multiple-comparisons test using Prism 5.0 software (GraphPad, San Diego, CA, USA). A *P*  value <0.05 was considered statistically significant.

## 3. Results

### 3.1. Curcumin Prevents K_**2**_Cr_**2**_O_**7**_-Induced Decrease in Body Weight Gain and Increase of Liver Weight and Liver/Body Ratio

**Figure 1:**
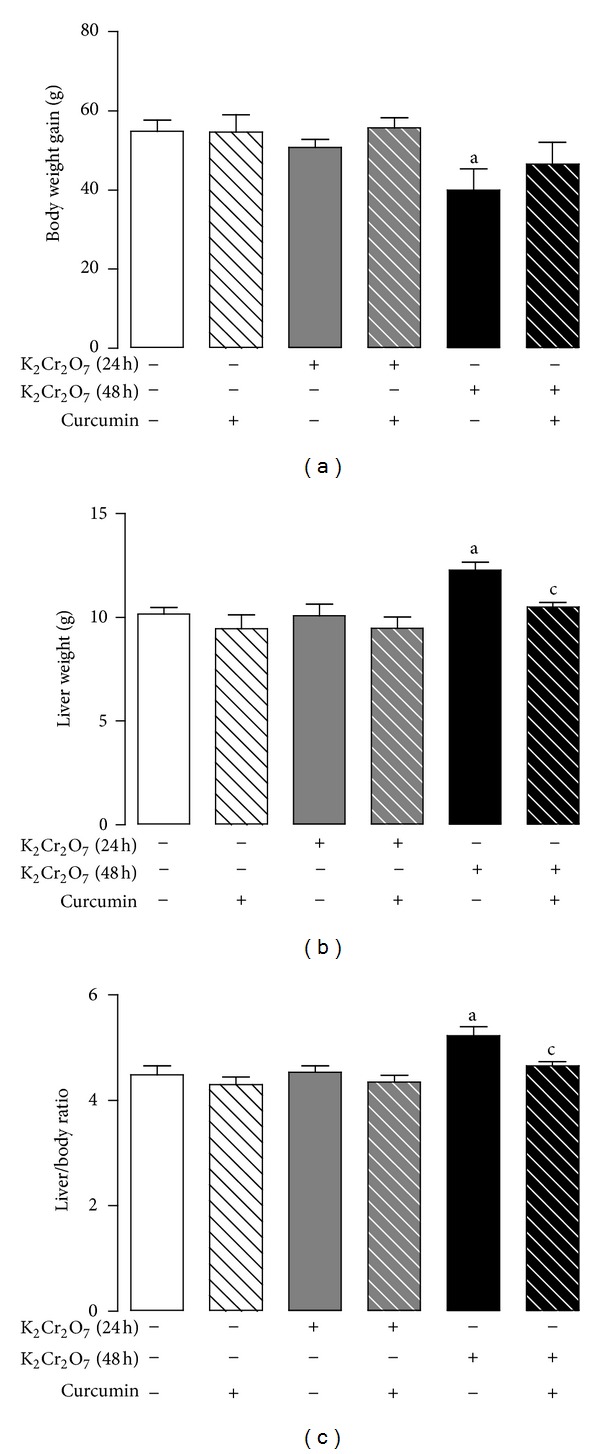
Effect of curcumin on body and liver weight of rats treated with K_2_Cr_2_O_7_. (a) Body weight gain, (b) liver weight, and (c) liver/body ratio. Values are mean ± SEM, *n* = 7-8. ^a^ *P* < 0.05 versus control; ^c^ *P* < 0.05  versus K_2_Cr_2_O_7_ (48 h).

Treatment with K_2_Cr_2_O_7_ resulted in a significant decrease in body weight gain and a significant increase in liver weight and liver/body ratio at 48 h (Figure 1). Pretreatment with curcumin significantly prevented these effects (Figure 1). 

### 3.2. Curcumin Prevents the K_**2**_Cr_**2**_O_**7**_-Induced Increase in the Plasma Activity of ALT, AST, LDH, and ALP

**Figure 2:**
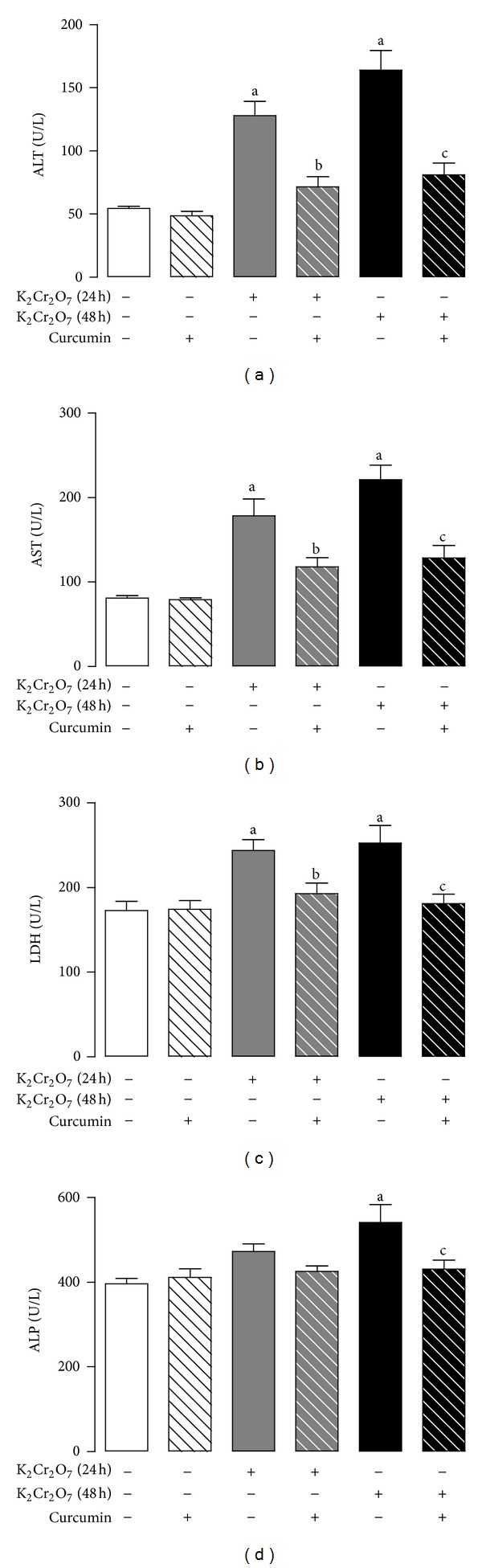
Effect of curcumin on the activity of (a) alanine aminotransferase (ALT), (b) aspartate aminotransferase (AST), (c) lactate dehydrogenase (LDH), and (d) alkaline phosphatase (ALP) in plasma of rats treated with K_2_Cr_2_O_7_. Values are mean ± SEM, *n* = 5–8. ^a^ *P* < 0.05 versus control; ^b^ *P* < 0.05  versus K_2_Cr_2_O_7_ (24 h);^c^ *P* < 0.05  versus K_2_Cr_2_O_7_ (48 h).

Rats treated with K_2_Cr_2_O_7_ exhibited a significant increase in plasma AST, ALT, and LDH activities at 24 and 48 h compared to control (Figure 2). Curcumin pretreatment significantly prevented the increase in the activity of AST, ALT, and LDH (Figure 2). The K_2_Cr_2_O_7_-induced increase in the activity of ALP at 48 h was prevented by curcumin (Figure 2).

### 3.3. Curcumin Prevents the K_**2**_Cr_**2**_O_**7**_-Induced Histological Damage

**Figure 3:**
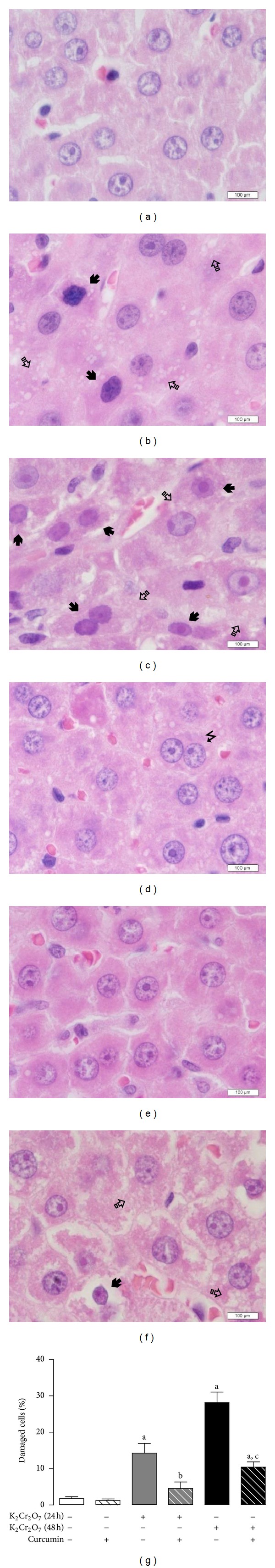
Representative histopathology in rats treated with curcumin, K_2_Cr_2_O_7_—or both. (a) Control animal that received only the vehicle show normal hepatic architecture. (b) Rat liver section after one day or two days (c) of K_2_Cr_2_O_7_ administration; there are injured hepatocytes with nuclear condensation (pyknosis) and cytoplasmic vacuolization (arrows), as well as regenerative binucleated hepatocytes. (d) In contrast, administration of curcumin alone did not produce histological damage. (e) Rat treated with curcumin after one day of K_2_Cr_2_O_7_ administration shows normal liver histology. (f) Liver section after two days of K_2_Cr_2_O_7_ administration in animal pretreated with curcumin exhibited mild cytoplasmic vacuolation without pyknosis and binucleated regenerative hepatocytes (all micrographs H/E, magnification 1000X). (g) Quantitative morphometry shows significant liver protection in groups pretreated with curcumin. Values are mean ± SEM, *n* = 3-4.  ^a^ *P* < 0.05 versus control; ^b^ *P* < 0.05 versus K_2_Cr_2_O_7_ (24 h); ^c^ *P* < 0.05  versus K_2_Cr_2_O_7_ (48 h).

Control and curcumin-treated groups presented normal hepatic structure, characterized by polygonal-shape hepatocytes with well-defined boundaries, slight staining acidophilic cytoplasm with large and centrally located nucleus with dispersed chromatin; some binucleated cells were also observed (Figures 3(a) and 3(d)). Treatment with K_2_Cr_2_O_7_ generated focal centrolobular hepatocytes death 24 h and 48 h, in a time-dependent fashion (Figures 3(b) and 3(c)); these cells showed extensive cytoplasmic vacuolation with pyknotic nucleus. In contrast, curcumin-pretreated groups showed almost normal histology; only the CUR-K_2_Cr_2_O_7_ group at 48 h presented occasional injured hepatocytes (Figures 3(e) and 3(f)). These features were confirmed by the quantification of damaged hepatocytes, which revealed that curcumin pretreatment prevented the K_2_Cr_2_O_7_-induced significant increase in hepatocytes damage at 24 and 48 h of about 15% and 30%, respectively (Figure 3(g)).

### 3.4. Curcumin Ameliorates K_**2**_Cr_**2**_O_**7**_-Induced Liver Oxidative Damage

**Figure 4:**
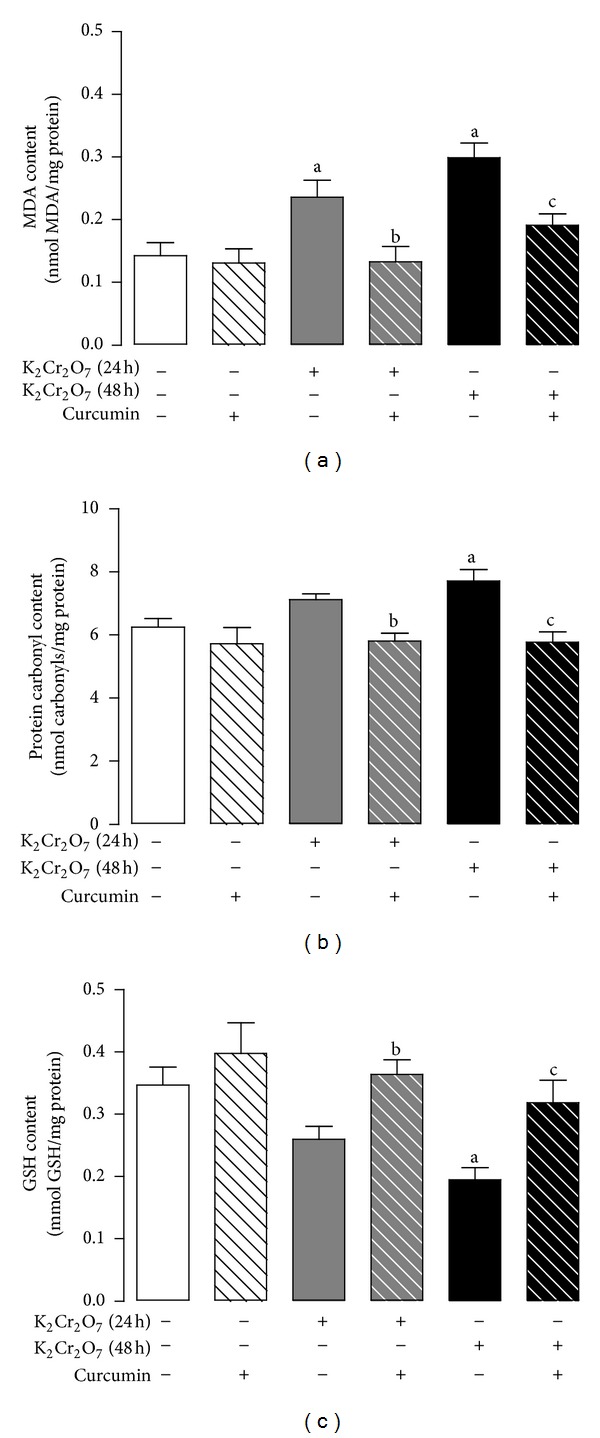
Effect of curcumin on the liver content of oxidative stress markers of rats treated with K_2_Cr_2_O_7_. (a) MDA content, (b) protein carbonyl content, and (c) GSH content. Values are mean ± SEM, *n* = 5-6. ^a^ *P* < 0.05  versus control; ^b^ *P* < 0.05  versus K_2_Cr_2_O_7_ (24 h); ^c^ *P* < 0.05  versus K_2_Cr_2_O_7_ (48 h).

Curcumin pretreatment prevents the K_2_Cr_2_O_7_-induced oxidative damage, which was made evident by the increase in the levels of MDA and protein carbonyl and a decrease in the levels of GSH at 48 h (Figure 4). In addition, K_2_Cr_2_O_7_ induced an increase of MDA levels at 24 h that was prevented by curcumin pretreatment (Figure 4). The K_2_Cr_2_O_7_-induced increase in protein carbonyl content and the decrease in GSH content at 24 h did not reach statistical significance (Figure 4).

### 3.5. Curcumin Prevents the K_**2**_Cr_**2**_O_**7**_-Induced Decrease in the Activity of Hepatic Antioxidant Enzymes

**Figure 5:**
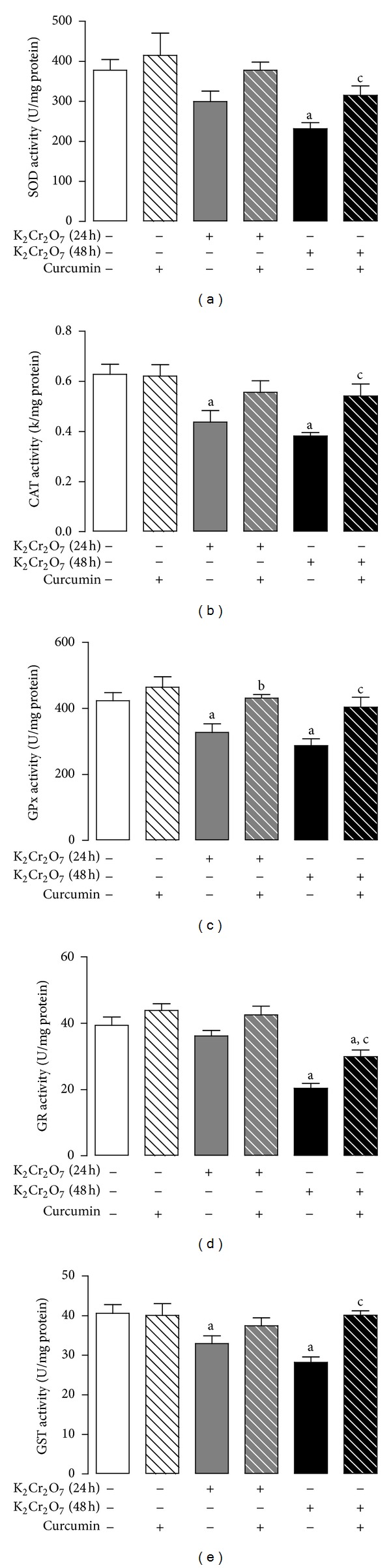
Effect of curcumin on liver activity of antioxidant enzymes activity of rats treated with K_2_Cr_2_O_7_. (a) Superoxide dismutase (SOD), (b) catalase (CAT), (c) glutathione peroxidase (GPx), (d) glutathione reductase (GR), and (e) glutathione-*S*-transferase (GST). Values are mean ± SEM, *n* = 5-6. ^a^ *P* < 0.05  versus control; ^b^ *P* < 0.05  versus K_2_Cr_2_O_7_ (24 h); ^c^ *P* < 0.05 versus K_2_Cr_2_O_7_ (48 h).

K_2_Cr_2_O_7_ reduced significantly the activity of the antioxidant enzymes SOD, CAT, GPx, GR, and GST at 48 h and that of GPx at 24 h that was prevented by curcumin (Figure 5). The K_2_Cr_2_O_7_-induced decrease in the activity of CAT and GST on 24 h was not prevented by curcumin pretreatment (Figure 5).

### 3.6. Curcumin Prevents K_**2**_Cr_**2**_O_**7**_-Induced Oxidative Damage and Decrease in the Activity of Antioxidant Enzymes in Isolated Hepatic Mitochondria

**Figure 6:**
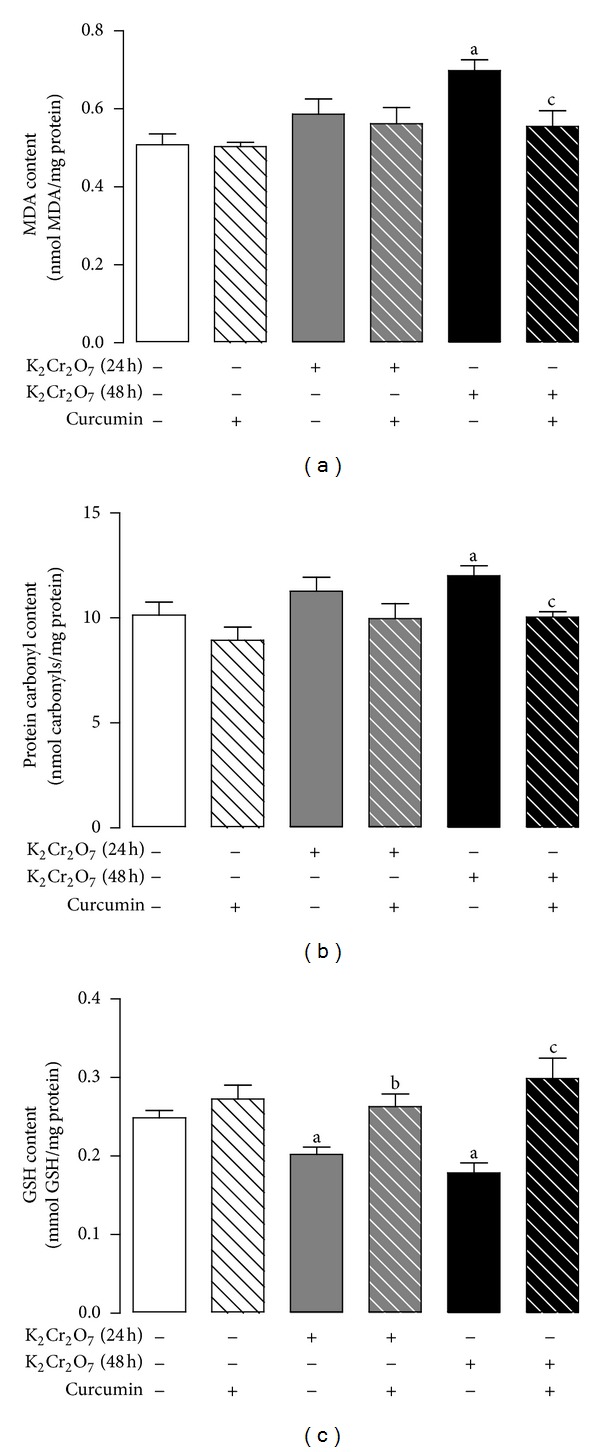
Effect of curcumin on the content of oxidative stress markers in hepatic mitochondria isolated from rats treated with K_2_Cr_2_O_7_. (a) MDA, (b) protein carbonyl, and (c) GSH. Values are mean ± SEM, *n* = 5-6. ^a^ *P* < 0.05  versus control; ^b^ *P* < 0.05  versus K_2_Cr_2_O_7_ (24 h); ^c^ *P* < 0.05  versus K_2_Cr_2_O_7_ (48 h).

**Figure 7:**
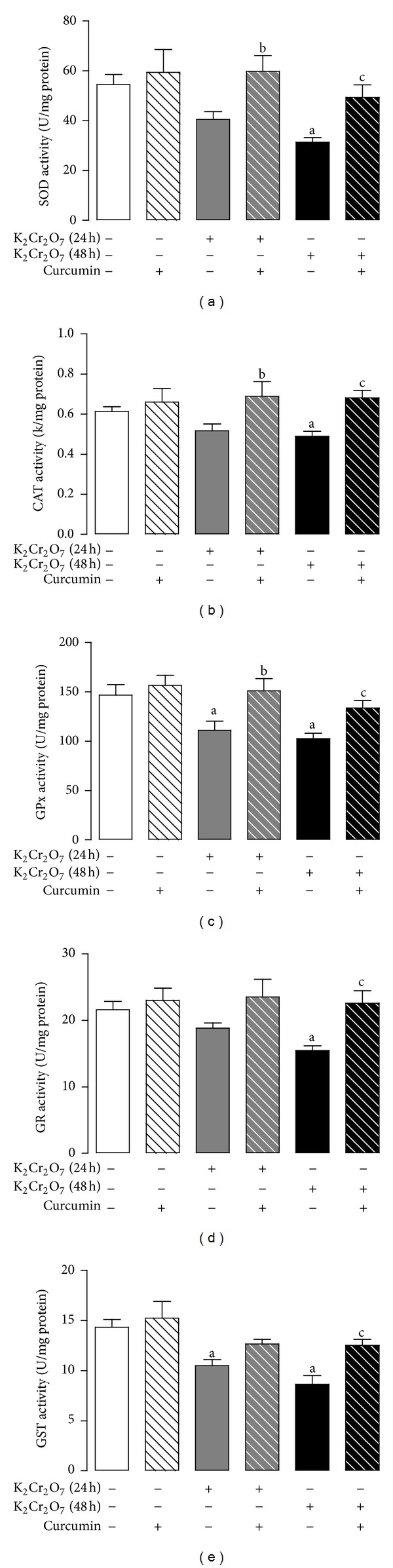
Effect of curcumin on the activity of antioxidant enzymes activity in hepatic mitochondria isolated from rats exposed to K_2_Cr_2_O_7_. (a) Superoxide dismutase (SOD), (b) catalase (CAT), (c) glutathione peroxidase (GPx), (d) glutathione reductase (GR), and (e) glutathione-*S*-transferase (GST). Values are mean ± SEM, *n* = 5-6. ^a^ *P* < 0.05  versus control; ^b^ *P* < 0.05  versus K_2_Cr_2_O_7_ (24 h); ^c^ *P* < 0.05  versus K_2_Cr_2_O_7_ (48 h).

Curcumin prevented the significant increase in K_2_Cr_2_O_7_-induced lipid peroxidation and protein carbonyl content in hepatic mitochondria at 48 h. Besides, K_2_Cr_2_O_7_ produced a significant decrease of GSH content at 24 and 48 h. These changes were effectively prevented by curcumin pretreatment (Figure 6). In addition, curcumin pretreatment prevented the K_2_Cr_2_O_7_-induced decrease in the activity of GPx and GST at 24 h and that of SOD, CAT, GPx, GR, and GST at 48 h (Figure 7).

### 3.7. Curcumin Protects against Mitochondrial Dysfunction Induced by K_**2**_Cr_**2**_O_**7**_

**Figure 8:**
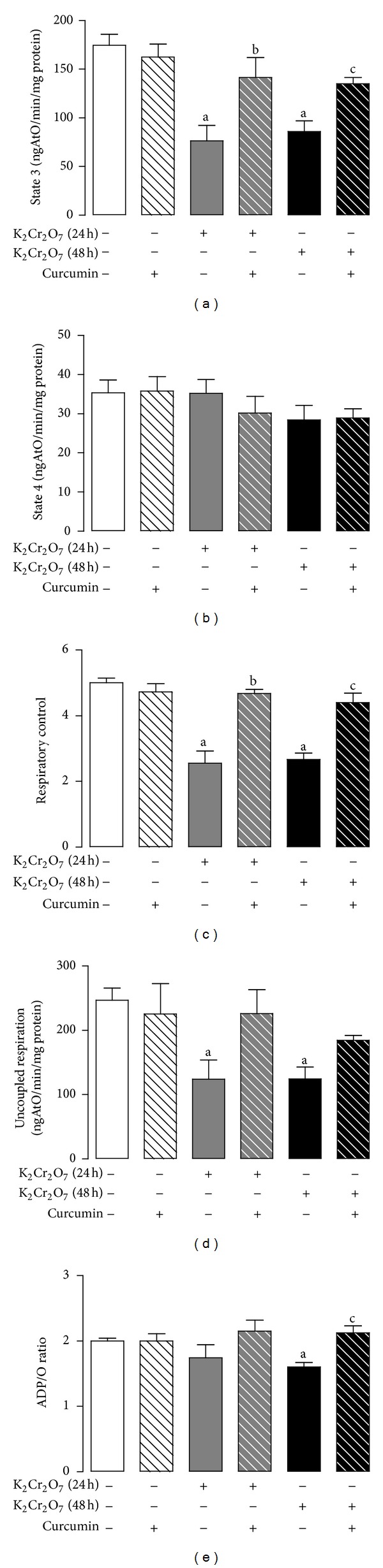
Curcumin prevents K_2_Cr_2_O_7_-induced alterations in mitochondrial oxygen consumption using malate/glutamate as substrate. CUR: curcumin; RCI: respiratory control index. Values are mean ± SEM, *n* = 4-5. ^a^ *P* < 0.05  versus control; ^b^ *P* < 0.05  versus K_2_Cr_2_O_7_ (24 h); ^c^ *P* < 0.05  versus K_2_Cr_2_O_7_ (48 h).

Curcumin prevented the K_2_Cr_2_O_7_-induced decrease in mitochondrial respiration evaluated by state 3 and respiratory control index at 24 h and 48 h using malate/glutamate as substrate (Figure 8). State 4 of respiration remained unchanged in all the studied groups (Figure 8). Curcumin also prevented the K_2_Cr_2_O_7_-induced decrease in the ADP/O ratio at 48 h (Figure 8). The prevention by curcumin of K_2_Cr_2_O_7_-induced decrease in uncoupled respiration was not significative (Figure 8). The changes observed using succinate as substrate were less marked. No changes were observed at 24 h; at 48 h it was found that K_2_Cr_2_O_7_ induced a decrease in state 3 and in uncoupled respiration, which were not significantly prevented by curcumin (Table 1). No changes were observed in respiratory control index, ADP/O ratio, and in state 4 (Table 1). All these data led us to analyze the activity of respiratory complex I.

### 3.8. Curcumin Prevents the K_**2**_Cr_**2**_O_**7**_-Induced Decrease in the Respiratory Complex I Activity

**Figure 9:**
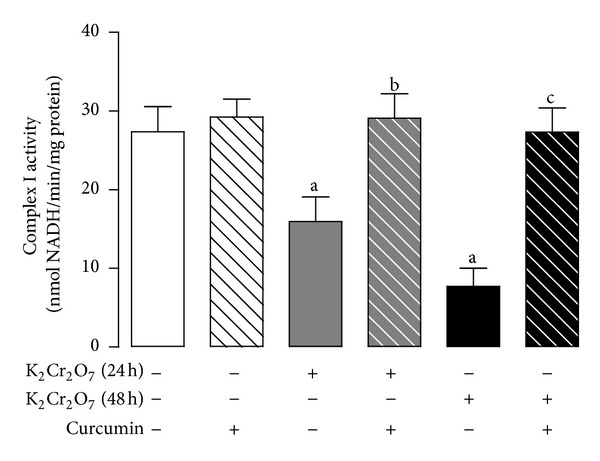
Curcumin prevents the K_2_Cr_2_O_7_-induced decrease in the activity of mitochondrial complex I. Values are mean ± SEM, *n* = 6-7. ^a^ *P* < 0.05  versus control; ^b^ *P* < 0.05 versus K_2_Cr_2_O_7_ (24 h); ^c^ *P* < 0.05  versus K_2_Cr_2_O_7_ (48 h).

K_2_Cr_2_O_7_ induced a significative decrease in the activity of respiratory complex I at 24 h and 48 h that was effectively prevented by curcumin pretreatment (Figure 9).

### 3.9. Curcumin Ameliorates the K_**2**_Cr_**2**_O_**7**_-Induced Membrane PTP Opening

**Figure 10:**
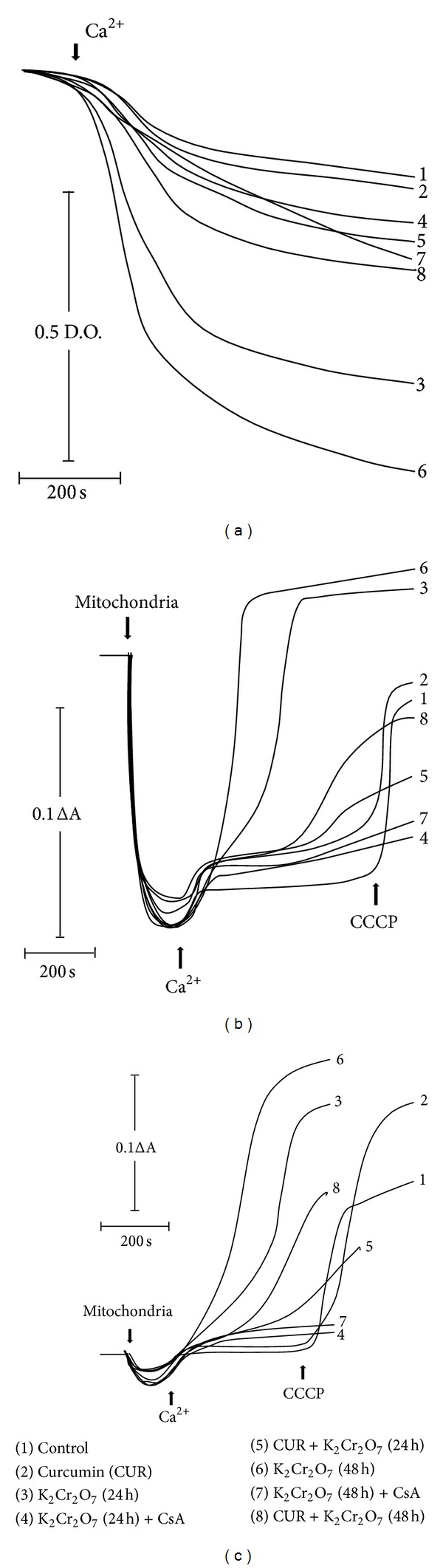
Effect of curcumin on the mitochondrial permeability transition pore-openining from rats exposed to K_2_Cr_2_O_7_. (a) Mitochondrial swelling, (b) mitochondrial membrane potential dissipation, and (c) mitochondrial Ca^2+^ retention. Tracings are representative of three different experiments. Carbonyl cyanide m-chlorophenylhydrazone (CCCP) was added in control and curcumin groups to induce loss of membrane potential and Ca^2+^ release. CUR: curcumin; CsA: cyclosporine A.

Curcumin reduced the K_2_Cr_2_O_7_-induced mitochondrial permeability transition determined by matrix swelling, membrane potential changes, and Ca^2+^ retention at 24 and 48 h. Isolated mitochondria from K_2_Cr_2_O_7_-treated rats presented a fast and considerable swelling after adding 50 *μ*M Ca^2+^ at 24 and 48 h. Curcumin pretreatment clearly avoided the K_2_Cr_2_O_7_-induced mitochondrial swelling (Figure 10(a)). In a similar way, K_2_Cr_2_O_7_ treatment sensitizes mitochondria to lose the membrane potential and the capacity of management Ca^2+^ by inducing PTP opening. On the contrary, curcumin pretreatment notably attenuates the K_2_Cr_2_O_7_-induced membrane potential collapse and Ca^2+^ release (Figures 10(b) and 10(c)). K_2_Cr_2_O_7_-induced PTP opening was prevented by CsA. Mitochondria from control or curcumin-treated rats did not present PTP opening even under conditions of Ca^2+^ overload until the protonophore CCCP was added.

### 3.10. Curcumin Prevents the K_**2**_Cr_**2**_O_**7**_-Induced Cyt C Release

**Figure 11:**
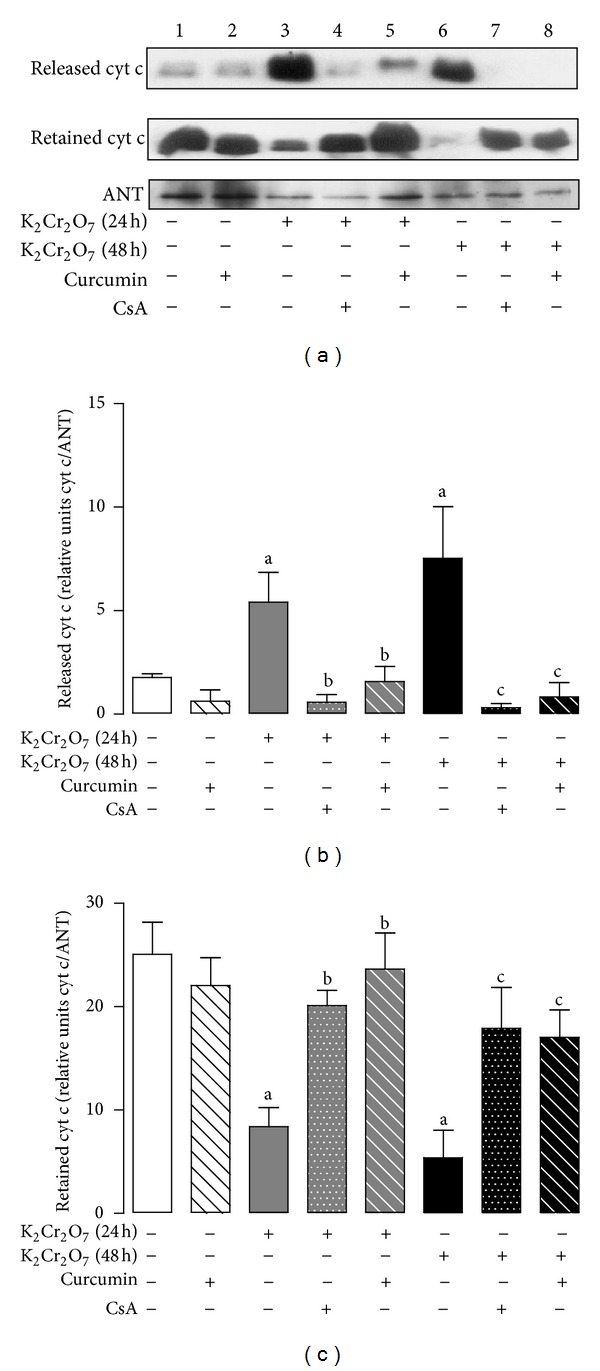
Effect of curcumin on cytochrome c (cyt c) release induced by the treatment with K_2_Cr_2_O_7_. Liver mitochondria were incubated for 10 min with 50 *μ*M Ca^2+^, in the presence or the absence of CsA. (a) Mitochondrial cyt c released and cyt c retained by Western blot analysis, (b) cyt c released/ANT ratio, and (c) cyt c retained/ANT ratio. Values are mean ± SEM, *n* = 3. ^a^ *P* < 0.05  versus control; ^b^ *P* < 0.05  versus K_2_Cr_2_O_7_ (24 h); ^c^ *P* < 0.05  versus K_2_Cr_2_O_7_ (48 h). ANT: adenine nucleotide translocator; CsA: cyclosporine A.

K_2_Cr_2_O_7_-induced PTP opening resulted in the release of proapoptotic factor cyt c to the cytosol at both 24 and 48 h (Figure 11(a), lanes 3 and 6). CsA abolished the K_2_Cr_2_O_7_-induced cyt c release (Figure 11(a), lanes 4 and 7). Interestingly, curcumin pretreatment prevents effectively the mitochondrial cyt c release that was induced by the treatment with K_2_Cr_2_O_7_ under conditions of Ca^2+^ overload (Figure 11(a), lanes 5 and 8). No effects were observed in control and curcumin groups (Figure 11(a), lanes 1 and 2). Densitometric analysis showed that released cyt c was significantly increased in mitochondria from K_2_Cr_2_O_7_-treated rats at 24 and 48 h. Curcumin and CsA prevented the cyt c release from mitochondria (Figure 11(b)). In contrast, retained cyt c in mitochondrial pellets from K_2_Cr_2_O_7_-treated rats was significantly diminished at both times while curcumin pretreatment and CsA maintained cyt c levels similar to control (Figure 11(c)). Together, these results indicate that K_2_Cr_2_O_7_ lowers the calcium-induced threshold for PTP opening induction and cytochrome c release.

## 4. Discussion

Experimental models of toxic liver injury are utilized to evaluate the biochemical processes involved in many forms of liver disease and to evaluate the possible pharmacological effects of candidate hepatoprotectants like curcumin [4].

Our data clearly show that curcumin pretreatment effectively prevented K_2_Cr_2_O_7_-induced hepatotoxicity. This protective effect was associated to the prevention of K_2_Cr_2_O_7_-induced oxidative damage, decrease in the activity of antioxidant enzymes in both liver homogenates and isolated mitochondria, and impairment in mitochondrial oxygen consumption, respiratory complex I inhibition, and PTP opening. The K_2_Cr_2_O_7_-induced hepatotoxicity was evident by the decrease in body weight gain, liver weight, and liver/body ratio, the increase in the plasma activity of ALT, AST, LDH, and ALP, and by the histopathological alterations. Kumar and Roy [70] also found that chromium induced a decrease in body weight gain, liver weight, and liver/body ratio. Increased plasma activity of ALT, AST, and ALP is indicative of hepatocellular damage since the disruption of the plasma membrane leak intracellular enzymes into the bloodstream [71, 72]. Treatment with K_2_Cr_2_O_7_ significantly augmented the activity of these enzymes in a time-dependent fashion. LDH in plasma is a presumptive marker of necrotic lesions in the hepatocytes [73]. Pretreatment with curcumin prevented the increase in the above-mentioned alterations, demonstrating the hepatoprotective effect of curcumin against the K_2_Cr_2_O_7_-induced damage. These findings are compatible with the results of other studies using curcumin against iron-induced hepatic toxicity [74] or thioacetamide-induced hepatic fibrosis [75]. Histopathological abnormalities were observed in liver of rats treated with K_2_Cr_2_O_7_ in a time-dependent fashion that correspond with the increase in the activity of plasma enzymes. The redox alterations caused by oxidative agents like Cr(VI) compounds have been shown to induce apoptosis and necrosis in hepatocytes and other cells [76]. In this way, the antioxidant curcumin prevented the K_2_Cr_2_O_7_-induced structural injury, preserving the normal architecture in liver tissue and saving hepatocytes from ROS. Previous studies have shown that curcumin protects against liver histological changes induced by toxins as carbon tetrachloride [17], acetaminophen [77], or cypermethrin [78]. Liver, the primary organ involved in the xenobiotic metabolism, is particularly susceptible to injury, and many reports suggest that chromium is a hepatotoxin [79–82]. Chromium induced-hepatotoxicity may be attenuated by several compounds [83–85]. The antihepatotoxic effects of curcumin against liver injury are well recognized and attributed to its intrinsic antioxidant properties [86–88]. 

Cr(VI) induces oxidative stress through enhanced ROS production leading to genomic DNA damage and oxidative deterioration of lipids and proteins. ROS include superoxide anion radical O_2_^•−^, hydrogen peroxide (H_2_O_2_), and the highly reactive hydroxyl radical (^•^OH) [20]. Lipid peroxidation generates a wide variety of end products, including MDA, which is used as a marker of oxidative damage. MDA may damage membrane proteins and lipids [89]. However, the formation of oxidized proteins is one of the highlights of oxidative stress, and the carbonyl groups (aldehydes and ketones) are produced on protein side chains when they are oxidized [90]. The results obtained show clearly that K_2_Cr_2_O_7_ increased lipid peroxidation and oxidized proteins reflecting hepatic oxidative damage. Curcumin pretreatment could prevent K_2_Cr_2_O_7_-induced oxidative damage because it is considered a bifunctional antioxidant exerting direct effects by scavenging ^•^OH, O_2_^•−^, and peroxyl radicals [91] and indirect effects inducing the expression of antioxidant enzymes [92]. 

GSH is a tripeptide (L-**γ**-glutamyl-L-cysteinylglycine) responsible for protection against ROS and other reactive species and detoxification of endogenous and exogenous toxins of an electrophilic nature [93]. Depletion of GSH decreases the antioxidant capacity and leads to oxidative stress [94, 95]. Rats treated with K_2_Cr_2_O_7_ presented low GSH levels in comparison with control, probably due to the oxidative stress induced for the K_2_Cr_2_O_7_ exposition. Soudani et al. [42] stated that this reduction in GSH levels might be due to its consumption in the scavenging of free radicals generated by K_2_Cr_2_O_7_. Curcumin pretreatment restored GSH levels, in a similar way to previous reports demonstrating the effectiveness curcumin in the reestablishment of the GSH content in the liver of rats exposed to paracetamol [14] or aflatoxin [96].

Antioxidant enzymes are important protective mechanisms against ROS. SOD catalyses the dismutation of O_2_^•−^ to O_2_ and to the less reactive species H_2_O_2_. Peroxides can be degraded by CAT or GPx [97]. GR converts GSSG to GSH by using NADPH whereas GST catalyzes the conjugation of electrophilic species with GSH [98]. In this study, K_2_Cr_2_O_7_ decreased the activity of SOD, CAT, GPx, GR, and GST mainly 48 h after treatment. This effect may be secondary to decreased enzyme levels (secondary to changes in synthesis or degradation of enzymes) or decreased activity (e.g., by oxidative damage) without changes in enzymes levels [95]. In this context, Kalayarasan et al. [73] postulated that K_2_Cr_2_O_7_ produces high levels of O_2_^•−^ which override enzymatic activity in liver tissues. Pretreatment with curcumin reestablished the activity of antioxidant enzymes to normal in animals exposed to K_2_Cr_2_O_7_, as has been shown in different curcumin hepatoprotection studies against sodium arsenite [99], acrylonitrile [100], chloroquine [101], or arsenic trioxide [102] toxicity. Besides, Iqbal et al. [5] revealed that curcumin administration increases several cytoprotective enzymes, especially in the liver.

Hepatocytes are normally rich in mitochondria, and each hepatocyte contains about 800 mitochondria occupying about 18% of the entire liver cell volume [103]. Mitochondria are targets of metal toxicity, and in many cases is related with oxidative stress and mitochondrial dysfunction [104–107]. Mitochondria are the main intracellular source of ROS as byproducts of the consumption of molecular oxygen in the electron transport chain and themselves are susceptible to oxidation; however, they possess a very effective antioxidant system [108, 109]. The experimental results in hepatic mitochondria isolated from rats treated with K_2_Cr_2_O_7_ demonstrate an increase in mitochondrial lipids and oxidized proteins, GSH depletion and reduction in the activity of antioxidant enzymes being this effect more consistent after 48 h of exposition. These results are in relation with previous studies [110, 111]. Moreover, curcumin successfully prevents mitochondrial oxidative damage and the alterations in the antioxidant enzyme activities caused by K_2_Cr_2_O_7_. Curcumin can protect rat liver mitochondria from ROS-induced lipid peroxidation and protein oxidation by donating H-atoms from its phenolic and methylenic groups of the *β*-diketone moiety [86, 112], augmenting levels of GSH [113, 114] or increasing the cytoprotective defenses, in a similar way with other experimental models [115, 116].

Oxidative stress leads to mitochondrial dysfunction, decrease in oxygen consumption and ATP production, alterations in calcium homeostasis, oxidation of DNA, proteins, and lipids, PTP opening, modifying of the expression of antioxidant enzymes and enhancing expression, and/or DNA binding of numerous transcription factors [117–119]. Our results showed that liver mitochondria isolated from rats exposed to K_2_Cr_2_O_7_ presented alterations in oxygen consumption by decreasing state 3 respiration, RCI, uncoupled respiration and ADP/O ratio using malate/glutamate. Negative effects using succinate were less evident, affecting state 3 and uncoupled respiration. These results indicate that K_2_Cr_2_O_7_ could damage biomolecules from the electron transport chain, especially complex I. In agreement, Cr(VI) has shown noxious effects on liver mitochondrial bioenergetics, as a consequence of its oxidizing activity which shunts electrons from electron donors coupled to ATP production, and to the ability of Cr(III), derived from Cr(VI) reduction, to form stable complexes with ATP precursors and enzymes involved in the ATP synthesis [46]. Noticeably, pretreatment with curcumin restored oxygen consumption supporting state 3 respiration, RCI, uncoupled respiration, and ADP/O ratio, suggesting well-coupled mitochondria. Curcumin prevents mitochondrial dysfunction by maintaining redox homeostasis or by protecting the mitochondrial respiratory complex [120, 121]. 

The mitochondrial respiratory chain is one of the major sources of damaging free radicals in human organism, and unpaired electrons escaping from the respiratory complexes (mainly from complexes I and III) can lead to the formation of O_2_^•−^ by the interaction with O_2_ [122]. Mitochondrial complex I is a large enzyme complex of over 40 subunits embedded in the inner mitochondrial membrane and has a central role in energy production by the mitochondrial respiratory chain, providing about 40% of the proton-motive force required for the synthesis of ATP [123]. Complex I dysfunction is the most common mitochondrial defect leading to cell death and disease [124]. The present findings show that K_2_Cr_2_O_7_ inhibited complex I activity in a time-dependent mode. Fernandes et al. [47] showed that in rat liver mitochondria complex I was more sensitive to Cr(VI) than complex II, and the activity of cytochrome c oxidase (complex IV) was not affected. Instead, curcumin pretreatment preserved the complex I activity possibly because curcumin scavenges ROS or reduces ROS production in complex I. Recently, it has been reported that curcumin attenuates the inhibition of mitochondrial complex I in K_2_Cr_2_O_7_-induced nephrotoxicity [50], peroxynitrite-induced neurotoxicity [125], or catecholamine-induced cardiotoxicity [126].

The transfer of electrons along the respiratory chain generates an electrochemical gradient across the mitochondrial inner membrane comprising both a membrane potential and H^+^ gradient [127]. In order to maintain those gradients, it is essential that the inner membrane of mitochondria remains impermeable or selectively permeable to metabolites and ions under normal aerobic conditions. However, in response to stress, the permeability of the mitochondrial membrane may increase, with the formation of a voltage-dependent nonspecific pore in the inner membrane known as the mitochondrial PTP [128]. PTP opening causes massive swelling of mitochondria, membrane depolarization, calcium release, rupture of the outer membrane, and release of intermembrane components that induce apoptosis [129]. Thus, the results confirmed that mitochondria from livers of K_2_Cr_2_O_7_-treated rats presented PTP opening and cyt c release, as previously described by Xiao et al. [130]. in L-02 hepatocyte and Pritchard et al. [131] in Chinese hamster ovary (CHO) cells. Curcumin pretreatment ameliorates the mitochondrial PTP opening from K_2_Cr_2_O_7_-treated rats protecting them from the noxious effects generated from Cr(VI). Besides, curcumin presents antihepatotoxic effects against ethanol-induced cytochrome c translocation in cultures of isolated rat hepatocytes [132], induces protective effects against catecholamine-induced cardiotoxicity by preserving mitochondrial function [126], and prevents mitochondrial dysfunction in an aging model [133]. 

Antihepatotoxic effects of curcumin were able to inhibit the PTP opening, and this outcome was related to its antioxidant properties by suppressing both O_2_^•−^ production and lipid peroxidation [134]. In contrast, antihepatocarcinogenic effects of curcumin induces the apoptosis of tumor cells through mitochondria-dependant pathways, including the release of cyt c, changes in electron transport, and loss of mitochondrial transmembrane potential as has been described in human hepatocellular carcinoma J5 cells [135, 136], and HepG2 cells [137, 138], by its potent antioxidant as well as anti-inflammatory properties [139]. Thus, curcumin has a dual effect inducing PTP opening.

In summary, acute K_2_Cr_2_O_7_-exposure enhances the oxidative stress both by mitochondrial dysfunction as well as due to the failure in the ROS removal system that in turn causes liver injury. Curcumin pretreatment successfully attenuated hepatic damage and prevented oxidative stress and the decrease in the activity of antioxidant enzymes in both liver homogenates and isolated mitochondria. Also, curcumin ameliorated respiratory complex I activity and avoided PTP opening. All these beneficial effects of curcumin protected liver from K_2_Cr_2_O_7_-induced hepatotoxicity associated with mitochondrial dysfunction.

## Figures and Tables

**Table 1:** Effect of curcumin pretreatment on K_2_Cr_2_O_7_-induced alterations in mitochondrial oxygen consumption using succinate as substrate.

	Control	CUR	K_2_Cr_2_O_7_ (24 h)	CUR-K_2_Cr_2_O_7 _ (24 h)	K_2_Cr_2_O_7_ (48 h)	CUR-K_2_Cr_2_O_7_ (48 h)
Succinate						
State 3 (ngAtO/min/mg protein)	243 ± 24	192 ± 9	169 ± 24	170 ± 36	156 ± 13^a^	180 ± 27
State 4 (ngAtO/min/mg protein)	59 ± 5	48 ± 2	51 ± 7	40 ± 10	49 ± 4	51 ± 5
RCI	4.1 ± 0.3	4.0 ± 0.2	3.5 ± 0.1	4.3 ± 0.2	3.2 ± 0.3	3.5 ± 0.4
Uncoupled respiration (ngAtO/min/mg protein)	396 ± 34	363 ± 21	284 ± 39	269 ± 63	245 ± 34^a^	269 ± 18
ADP/O	1.3 ± 0.1	1.6 ± 0.1	1.6 ± 0.1	1.6 ± 0.2	1.4 ± 0.2	1.4 ± 0.1

Values are mean ± SEM, *n* = 4-5. CUR: curcumin; RCI: respiratory control index. ^a^ *P* < 0.05 versus control.
